# Monthly Trends in Access to Care and Mental Health Services by Household Income Level During the COVID-19 Pandemic, United States, April: December 2020

**DOI:** 10.1089/heq.2021.0036

**Published:** 2021-11-09

**Authors:** Hyunjung Lee, Gopal K. Singh

**Affiliations:** ^1^ Oak Ridge Institute for Science and Education (ORISE), Oak Ridge, Tennessee, USA.; ^2^ US Department of Health and Human Services, Health Resources and Services Administration, Office of Health Equity, Rockville, Maryland, USA.

**Keywords:** access to care, COVID-19, delayed medical care due to pandemic, mental health services, income inequalities, trend

## Abstract

**Purpose:** Since the start of the coronavirus disease 2019 (COVID-19) pandemic in March 2020, ∼40% of U.S. adults have experienced delayed medical care. Rates of uninsurance, delayed care, and utilization of mental health services during the course of the pandemic have not been analyzed in detail. We examined monthly trends and disparities in access to care by household income levels in the United States.

**Methods:** Using Census Bureau's nationally representative pooled 2020 Household Pulse Survey from April to December, 2020 (*N*=778,819), logistic regression models were used to analyze trends and inequalities in various access to care measures.

**Results:** During the COVID-19 pandemic, the odds of being uninsured, having a delayed medical care due to pandemic, delayed care of something other than COVID-19, or delayed mental health care were, respectively, 5.54, 1.50, 1.85, and 2.18 times higher for adults with income <$25,000, compared to those with incomes ≥$200,000, controlling for age, sex, race/ethnicity, education, marital status, housing tenure, region of residence, and survey month. Income inequities in mental health care widened over the course of the pandemic, while the probability of delayed mental health care increased for all income groups. Although the odds of taking prescription medication for mental health were higher for low-income adults, the odds of receiving mental health services were generally lower for lower income adults, controlling for all covariates.

**Conclusion:** In light of our findings on persistent health care inequities during the pandemic, increased policy efforts are needed to improve access to care in low-income populations as an equitable COVID-19 recovery response.

## Introduction

Since the coronavirus disease 2019 (COVID-19) pandemic began in March, there has been delayed or forgone medical health care during the pandemic.^1–6^ Approximately 40% of adults aged ≥18 years have foregone medical care during the pandemic^2,3^ and 20% of households have been unable to get medical care for serious problems since the start of the coronavirus outbreak.^4^ Approximately 21% of Medicare beneficiaries were not able to get health care for something other than COVID-19 during the 2020 summer^5^ and 8% of beneficiaries during the 2020 fall.^6^ Hospitals experienced a reduction in the average numbers of diagnosed cases of some medical emergencies such as acute myocardial infarction and ischemic stroke cases^7^ and had a decrease in the non-COVID-19 hospitalizations^8^ during the COVID-19 pandemic.

Improving access to health services, including a reduction in unmet health needs or delays in receiving appropriate care, and understanding social determinants of health have long been the objective toward achieving health equity for all Americans.^9,10^ Delayed access or a lack of access to health care has been associated with negative health outcomes, for example, potential comorbidities or poor quality of life,^11,12^ a missed diagnosis and progress of disease,^13^ higher mortality, lower life expectancy, or poor self-reported health status.^12,14,15^ Delayed or nonreceipt of needed medical care and prescription drugs have been more prevalent among vulnerable populations such as low-income individuals or racial/ethnic minorities for decades in the United States.^16,17^ During the pandemic, disproportionately high percentage of individuals with low-income presented having serious financial problems and job or wage losses, and the use of public transportation.^4^ These financial difficulties and limited options of transportation among low-income group might exacerbate delayed care due to closing or limited services of doctor's office or facilities, unsafety concerns, and cost concerns.^2^ The recent Presidential Executive Order emphasized assessing whether personal protective equipment, tests, vaccines, therapeutics, and other resources have been or will be allocated equitably by considering barriers that have restricted access to preventive measures, treatment, and other health services for high-risk populations.^18^

Previous research has examined the association between access to care during the pandemic and household income level, but with mixed findings. Hamel et al., using a survey in June, 2020, found that individuals with income less than $40,000 were less likely to report delaying care due to coronavirus than those with income more than $40,000.^2^ Czeisler and colleagues using another survey data in June, 2020, found no statistically significant differences in delayed care during the COVID-19 pandemic across income groups.^3^ However, one report, using a survey in July, 2020, found that households with incomes below $30,000 were more likely to have serious problems affording medical care during the pandemic, compared to those with incomes more than $30,000.^4^ To fill the gap in the existing literature particularly related to health care inequities during the pandemic, we examined monthly trends in uninsured rate, delayed care, and utilization of mental health services by household income level and estimated the association between access measures and household income level among adults aged ≥18 years in the United States, using a nationally representative dataset from April to December 2020.

## Methods

### Data

The data for this study are derived from the 2020 Household Pulse Survey (HPS), a nationally representative online survey developed by the U.S. Census Bureau with other federal agencies.^19^ The HPS utilized the Census Bureau's Master Address File as a source of sample, enabling to estimate at three different geographical levels, including Metropolitan Statistical Areas, state level, and national level.^19^ The HPS, as a short-turnaround instrument, provides employment status, spending patterns, food security, housing, physical and mental health, access to health care, and educational disruption among households in the United States during the COVID-19 pandemic.^19^ The HPS was initially developed on a weekly basis with three times of interviews for the same household in Phase 1, but Phase 2 and Phase 3 of the HPS consisted of 2-week cross-sectional datasets.^19^ For this study, to estimate monthly trend, we appended data of the weeks 1, 4, 7, and 10 from Phase 1, and weeks 13, 15, and 17 from Phase 2, and 19 and 21 from Phase 3, containing 9 months of HPS data during the pandemic from April to December, 2020. Human Participant Protection: The study was exempt from Institutional Review Board approval as it utilized a de-identified public use dataset.

### Sample

The study sample comprised adults aged 18 to 88 in the 2020 HPS from April to December. The final sample size varied by the outcome measure ranging from 366,183 to 674,381 (Table 1) due to missing values, although the pooled sample size was 778,819. We created missing covariate categories to prevent omission of many observations from the analysis for income (6.11%), marital status (0.42%), and housing tenure (1.69%).

**Table 1.** Weighted Proportions of Outcome Measures and Individual Characteristics by Household Income Level Among U.S. Adults Aged ≥18 Years, April–December 2020 Household Pulse Survey

% (SE)	Overall	Less than $25,000	$25,000–$49,999	$50,000–$99,999	$100,000–$199,999	$200,000 and above
Uninsured rate (*n*=674,381)	9.07 (0.10)	17.80 (0.40)	13.76 (0.27)	7.26 (0.16)	2.84 (0.12)	1.40 (0.15)
Delayed medical care due to pandemic (*n*=673,352)	36.63 (0.14)	39.30 (0.48)	36.50 (0.33)	37.18 (0.25)	36.33 (0.26)	34.58 (0.39)
Delayed care of something other than COVID-19 (*n*=673,803)	28.29 (0.13)	33.95 (0.46)	29.39 (0.30)	28.87 (0.24)	25.46 (0.23)	22.59 (0.36)
Delayed mental health care (*n*=366,399)	10.74 (0.11)	15.74 (0.44)	12.17 (0.26)	10.78 (0.20)	8.61 (0.19)	6.34 (0.27)
Taking prescription medication for mental health (*n*=366,228)	20.55 (0.14)	26.66 (0.51)	21.43 (0.32)	20.63 (0.23)	18.77 (0.24)	15.94 (0.36)
Receiving mental health service (*n*=366,183)	9.57 (0.10)	13.24 (0.42)	8.64 (0.21)	8.91 (0.16)	9.41 (0.17)	10.69 (0.33)
Individual characteristics (*n*=674,381)						
Age (years)						
18–24	7.22 (0.10)	24.81 (0.67)	24.38 (0.65)	20.75 (0.63)	12.73 (0.49)	4.44 (0.28)
25–34	18.48 (0.12)	15.94 (0.32)	25.26 (0.34)	30.64 (0.33)	17.40 (0.25)	4.15 (0.13)
35–44	17.68 (0.11)	12.15 (0.26)	19.52 (0.28)	28.44 (0.28)	24.40 (0.24)	8.60 (0.14)
45–54	16.65 (0.10)	11.58 (0.25)	18.51 (0.30)	27.96 (0.30)	25.44 (0.26)	10.13 (0.15)
55–64	17.87 (0.11)	12.74 (0.28)	20.10 (0.30)	29.88 (0.30)	23.03 (0.26)	8.47 (0.14)
65–74	15.97 (0.11)	12.70 (0.30)	25.50 (0.34)	32.24 (0.35)	17.66 (0.23)	4.88 (0.12)
75+	6.13 (0.07)	13.44 (0.57)	28.41 (0.55)	29.83 (0.54)	14.50 (0.44)	4.54 (0.22)
Sex						
Male	48.16 (0.15)	11.43 (0.19)	20.91 (0.21)	29.71 (0.21)	22.75 (0.17)	8.13 (0.10)
Female	51.84 (0.15)	16.28 (0.17)	23.73 (0.17)	28.65 (0.17)	18.43 (0.13)	5.69 (0.07)
Race/ethnicity						
Non-Hispanic White	64.51 (0.16)	10.74 (0.12)	20.03 (0.14)	31.17 (0.15)	23.53 (0.13)	8.03 (0.07)
Non-Hispanic Black	10.79 (0.10)	23.98 (0.49)	28.15 (0.46)	24.54 (0.42)	12.02 (0.31)	2.47 (0.11)
Hispanic	15.84 (0.14)	20.43 (0.45)	29.59 (0.49)	25.65 (0.42)	12.70 (0.28)	3.38 (0.15)
Non-Hispanic Asian	5.04 (0.07)	9.71 (0.55)	16.24 (0.56)	25.74 (0.63)	26.28 (0.53)	14.08 (0.41)
Non-Hispanic other race	3.82 (0.06)	18.31 (0.63)	23.63 (0.59)	27.31 (0.66)	18.17 (0.54)	4.54 (0.22)
Education						
Less than high school	7.18 (0.13)	36.23 (0.90)	29.65 (0.87)	16.92 (0.68)	5.06 (0.36)	1.82 (0.24)
High school	29.79 (0.16)	19.45 (0.29)	30.34 (0.31)	29.01 (0.31)	11.15 (0.22)	1.72 (0.08)
Some college	30.68 (0.13)	13.52 (0.17)	24.64 (0.20)	32.54 (0.21)	18.71 (0.17)	3.54 (0.09)
Bachelor's degree	17.93 (0.08)	5.47 (0.11)	13.85 (0.16)	31.08 (0.21)	32.02 (0.21)	12.04 (0.15)
Master's degree or higher	14.42 (0.07)	2.91 (0.08)	8.03 (0.13)	25.99 (0.20)	37.02 (0.23)	20.67 (0.19)
Marital status						
Currently married	56.47 (0.15)	5.89 (0.12)	17.48 (0.16)	32.41 (0.18)	27.52 (0.15)	9.78 (0.09)
Widowed	4.28 (0.06)	25.30 (0.72)	34.66 (0.70)	22.36 (0.57)	7.60 (0.30)	2.15 (0.21)
Divorced/separated	14.25 (0.10)	25.66 (0.38)	30.13 (0.36)	25.79 (0.32)	10.18 (0.18)	2.36 (0.09)
Never married	24.58 (0.14)	23.65 (0.32)	26.91 (0.31)	24.95 (0.29)	12.74 (0.21)	3.62 (0.11)
Missing	0.42 (0.02)	15.12 (1.75)	25.32 (2.26)	22.74 (2.09)	14.05 (1.38)	5.96 (0.90)
Housing tenure (home ownership)						
Renter	30.43 (0.15)	28.10 (0.30)	31.25 (0.28)	24.22 (0.24)	9.74 (0.15)	2.30 (0.06)
Owner	67.41 (0.15)	7.89 (0.11)	18.99 (0.15)	32.25 (0.16)	25.98 (0.14)	9.14 (0.08)
Missing	2.16 (0.05)	3.55 (0.36)	2.70 (0.31)	2.32 (0.23)	1.58 (0.24)	0.51 (0.10)
Region of residence						
Northeast	17.12 (0.12)	11.81 (0.31)	19.06 (0.31)	29.39 (0.35)	23.24 (0.29)	9.06 (0.16)
South	37.82 (0.15)	15.70 (0.21)	23.86 (0.22)	28.55 (0.22)	18.66 (0.17)	5.75 (0.08)
Midwest	20.76 (0.11)	12.82 (0.21)	23.10 (0.26)	31.24 (0.26)	20.39 (0.20)	5.30 (0.09)
West	24.30 (0.13)	13.66 (0.29)	21.74 (0.29)	28.17 (0.27)	21.55 (0.23)	8.40 (0.14)

Differences in unadjusted weighted means by household income level were statistically significant (*p*<0.001), tested by chi-square test.

COVID-19, coronavirus disease 2019; SE, standard error.

### Outcome measurement

Six outcome measures on access to care were used, including uninsured rate, delayed medical care due to pandemic, delayed care of something other than COVID-19, delayed mental health care, taking prescription medication for mental health, and receiving mental health services. The latter three outcome measures on access to mental health services were available from August to December. Respondents were asked (1) current health insurance coverage, and at any time in the last 4 weeks, whether they (2) “delay getting medical care because of the coronavirus pandemic?”; (3) “need medical care for something other than coronavirus, but did not get it because of the coronavirus pandemic?”; (4) “need counseling or therapy from a mental health professional, but did not get it for any reason?”; (5) “take prescription medication to help you with any emotions or with your concentration, behavior, or mental health?”; and (6) “receive counseling or therapy from a mental health professional such as a psychiatrist, psychologist, psychiatric nurse, or clinical social worker?.” All outcome measures were dichotomized with 1 being yes and zero equaling no.

### Independent variable of interest

Household income was defined by six categories: less than $25,000; $25,000–$49,999; $50,000–$99,999; $100,000–$199,999; and $200,000 and above; missing. Although the original questionnaire has nine categories, we combined $25,000–$34,999, $35,000–$49,999 and $50,000–$74,999, $75,000–$99,999 to each category, given similar patterns in trend of access to care in the study sample.

### Covariates

Based on the previous literature and data availability, we selected the following covariates for model estimation: age, sex, race/ethnicity, education, marital status, housing tenure, region of residence, and survey month.^3,20–22^ These covariates were measured as shown in Table 1. Age at the baseline was defined by seven categories: 18–24, 25–34, 35–44, 45–54, 55–64, 65–74, and ≥75. Race/ethnicity was defined by six categories as non-Hispanic white, non-Hispanic black, Hispanic, non-Hispanic Asian, and non-Hispanic other races. Educational attainment was defined by five categories as less than high school diploma, high school diploma or GED, some college, bachelor's degree, and master's degree, or higher. Marital status was categorized as currently married, widowed, divorced/separated, never married, and missing. Housing tenure was categorized as homeowners, renters, and missing. Region of residence was defined by four categories, Northeast, Midwest, South, and West.

### Analytic approach

Logistic regression models were used to estimate the association between access to care and income level during the pandemic, controlling for individual characteristics, and month-fixed effects. Average marginal effects were calculated with delta-method standard errors. Complex survey design procedures were used to account for nonresponse, occupancy of the housing unit counts, the number of adults within the housing unit, and disproportionate sampling of demographic characteristics.^19^ The sample weights were adjusted by dividing by the number of pooling months. All analyses were conducted by Stata 16.^23^

## Results

### Descriptive statistics

**FIG. 1.**
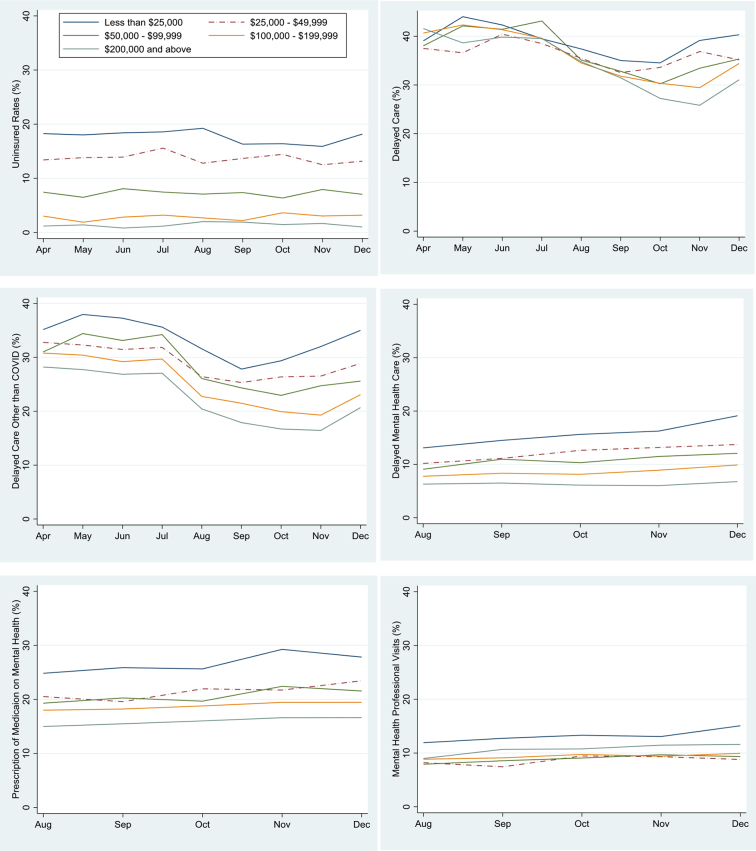
Trends in health care access by household income during the COVID-19 pandemic, April–December 2020 HPS. Figures show monthly trends in the weighted proportions of being uninsured (*n*=674,381), Delayed medical care due to pandemic (*n*=673,352), delayed care of something other than COVID-19 (*n*=673,803), delayed mental health care (*n*=366,399), taking prescription medication on mental health (*n*=366,228), and receiving mental health service (*n*=366,183) for adults aged 18 and older. Data derived from the April to December 2020 HPS. COVID-19, coronavirus disease 2019; HPS, Household Pulse Survey.

Figure 1 presents monthly trends in uninsured rate, delayed care, and utilization of mental health services among adults during the COVID-19 pandemic, from April to December, 2020. The graph of uninsured rates showed a slight drop and rise but remained relatively flat during the pandemic period, with marked differences by household income levels persisting across months. The rates of delayed care due to pandemic and delayed care of something other than COVID-19 increased during the first 2 months after the pandemic started especially for the lowest income group, had declined by September, but increased again since October. The probability of delayed mental health care increased for all income groups, but income inequities in mental health care widened over the course of the pandemic. The probability of taking prescription medication for mental health and receiving mental health service presented a marginally increasing trend, especially for the lowest-income group (less than $25,000) since August, but remained relatively unchanged for the other income groups.

Monthly trends of weighted unadjusted access measures for each group were statistically significant (*p*<0.05), except for the trend of uninsured rates for all income groups, and delayed mental health care and taking prescription medication for mental health for the highest income group (data not shown).

Table 1 provides weighted proportions of outcome measures and covariates by household income levels. Approximately 17.8% of adults with income less than $25,000 was uninsured, while 1.4% of adults with income ≥$200,000 was uninsured. Similarly, adults in the lowest-income category had higher rates of delayed care due to pandemic (39.3% vs. 34.6%), delayed care of something other than COVID-19 (34.0% vs. 22.6%), and delayed mental health care (15.7% vs. 6.3%) than those in the highest-income category. However, adults in the lowest-income category had higher rates of taking prescription medication for mental health (26.7% vs. 15.9%) and receiving mental health services (13.2% vs. 10.7%) than those in the highest income category.

The proportion of adults with income less than $25,000 was higher among the 18–24 age group, females, non-Hispanic blacks and Hispanics, adults with education less than high school, nonmarried adults, renters, or residents of the South compared with males, non-Hispanic whites, non-Hispanic Asians, those with a master's degree, currently married adults, homeowners, or residents of the Northeast.

### Logistic regression model results

Table 2 provides odds ratios of logistic regression estimates of the association between access to care and income level. The odds of being uninsured was 5.54 times higher for adults with household income less than $25,000 than those with income $200,000 and above, controlling for age, sex, race/ethnicity, education, marital status, housing tenure, region of residence, and survey month. The odds of delayed medical care due to pandemic, delayed care of something other than COVID-19, and delayed mental health care were, respectively, 1.50, 1.85, and 2.18 times higher for adults with household income less than $25,000 than those with income $200,000 and above, controlling for all other factors. The odds of taking prescription medication for mental health and receiving mental health services were, respectively, 1.9 and 1.3 times higher for adults with income less than $25,000 than those with income $200,000 and above, controlling for all covariates. Except for the lowest income group, the odds of receiving mental health services were lower for lower income group, while the odds of taking prescription medication for mental health were higher for lower income group, compared to those for the highest income group.

**Table 2.** Adjusted Odds Ratio of Access to Care by Household Income Level Among U.S. Adults Aged 18 Years or Older, April–December 2020 Household Pulse Survey

	Uninsured rate	Delayed medical care due to pandemic	Delayed care of something other than COVID-19	Delayed mental health care	Taking prescription medication for mental health	Receiving mental health service
Sample size	674,381	673,352	673,803	366,399	366,228	366,183
Household income						
Less than $25,000	5.54^***^ (0.67)	1.50^***^ (0.05)	1.85^***^ (0.06)	2.18^***^ (0.15)	1.90^***^ (0.08)	1.30^***^ (0.08)
$25,000–$49,999	5.18^***^ (0.62)	1.32^***^ (0.03)	1.52^***^ (0.04)	1.84^***^ (0.11)	1.46^***^ (0.06)	0.87^*^ (0.05)
$50,000–$99,999	3.35^***^ (0.39)	1.27^***^ (0.03)	1.45^***^ (0.04)	1.71^***^ (0.09)	1.36^***^ (0.04)	0.89^*^ (0.04)
$100,000–$199,999	1.61^***^ (0.19)	1.14^***^ (0.02)	1.18^***^ (0.03)	1.34^***^ (0.07)	1.19^***^ (0.04)	0.90^**^ (0.04)
$200,000 and above	1.00	1.00	1.00	1.00	1.00	1.00
Missing	3.77^***^ (0.48)	1.15^***^ (0.04)	1.32^***^ (0.05)	1.28^**^ (0.1)	1.16^**^ (0.06)	0.84^*^ (0.06)
Age (years)						
18–24	6.79^***^ (1.31)	1.16^**^ (0.05)	1.09 (0.06)	6.12^***^ (0.96)	1.36^***^ (0.1)	3.11^***^ (0.46)
25–34	9.93^***^ (1.86)	1.40^***^ (0.05)	1.33^***^ (0.05)	5.27^***^ (0.78)	1.41^***^ (0.08)	3.39^***^ (0.46)
35–44	8.45^***^ (1.59)	1.57^***^ (0.05)	1.55^***^ (0.06)	4.62^***^ (0.67)	1.63^***^ (0.09)	3.36^***^ (0.44)
45–54	6.97^***^ (1.31)	1.62^***^ (0.05)	1.70^***^ (0.06)	3.65^***^ (0.53)	1.76^***^ (0.09)	2.60^***^ (0.34)
55–64	4.63^***^ (0.88)	1.54^***^ (0.05)	1.54^***^ (0.06)	2.45^***^ (0.35)	1.62^***^ (0.08)	1.86^***^ (0.24)
65–74	0.58^*^ (0.12)	1.25^***^ (0.04)	1.17^***^ (0.04)	1.33^*^ (0.19)	1.31^***^ (0.07)	1.20 (0.15)
75+	1.00	1.00	1.00	1.00	1.00	1.00
Sex						
Male	1.00	1.00	1.00	1.00	1.00	1.00
Female	0.66^***^ (0.02)	1.31^***^ (0.02)	1.24^***^ (0.02)	1.67^***^ (0.04)	1.86^***^ (0.03)	1.51^***^ (0.04)
Race/ethnicity						
Non-Hispanic White	1.00	1.00	1.00	1.00	1.00	1.00
Non-Hispanic Black	0.98 (0.04)	0.87^***^ (0.02)	1.00 (0.02)	0.78^***^ (0.04)	0.47^***^ (0.02)	0.72^***^ (0.03)
Hispanic	1.75^***^ (0.06)	0.98 (0.02)	1.00 (0.02)	0.83^***^ (0.04)	0.61^***^ (0.02)	0.80^***^ (0.03)
Non-Hispanic Asian	1.02 (0.07)	0.79^***^ (0.02)	0.78^***^ (0.03)	0.45^***^ (0.03)	0.31^***^ (0.02)	0.45^***^ (0.03)
Non-Hispanic other race	0.94 (0.06)	1.20^***^ (0.04)	1.36^***^ (0.04)	1.22^**^ (0.08)	0.87^**^ (0.04)	1.02 (0.08)
Education						
Less than high school	4.24^***^ (0.28)	0.62^***^ (0.03)	0.80^***^ (0.03)	0.68^***^ (0.06)	0.78^***^ (0.05)	0.52^***^ (0.05)
High school	2.9^***^ (0.14)	0.62^***^ (0.01)	0.78^***^ (0.02)	0.65^***^ (0.03)	0.79^***^ (0.02)	0.43^***^ (0.02)
Some college	2.22^***^ (0.1)	0.85^***^ (0.01)	1.04^**^ (0.02)	0.96 (0.03)	1.00 (0.02)	0.67^***^ (0.02)
Bachelor's degree	1.35^***^ (0.06)	0.88^***^ (0.01)	0.91^***^ (0.01)	0.95 (0.03)	0.93^***^ (0.02)	0.82^***^ (0.02)
Master's degree or higher	1.00	1.00	1.00	1.00	1.00	1.00
Marital status						
Currently married	1.00	1.00	1.00	1.00	1.00	1.00
Widowed	1.36^**^ (0.15)	0.97 (0.03)	1.00 (0.04)	1.43^***^ (0.12)	1.21^***^ (0.05)	1.38^***^ (0.1)
Divorced/separated	1.49^***^ (0.06)	1.05^*^ (0.02)	1.08^***^ (0.02)	1.51^***^ (0.05)	1.31^***^ (0.03)	1.66^***^ (0.06)
Never married	1.51^***^ (0.05)	0.95^**^ (0.02)	0.90^***^ (0.02)	1.38^***^ (0.05)	1.10^***^ (0.03)	1.38^***^ (0.05)
Missing	1.09 (0.23)	1.27^*^ (0.13)	1.34^**^ (0.14)	1.24 (0.19)	1.05 (0.14)	1.25 (0.2)
Housing tenure (home ownership)						
Renter	1.41^***^ (0.04)	1.09^***^ (0.02)	1.12^***^ (0.02)	1.31^***^ (0.04)	1.15^***^ (0.03)	1.32^***^ (0.04)
Owner	1.00	1.00	1.00	1.00	1.00	1.00
Missing	1.24^*^ (0.11)	0.9 (0.06)	0.88 (0.06)	0.93 (0.16)	0.99 (0.08)	1.19 (0.14)
Region of residence						
Northeast	0.83^***^ (0.04)	0.95^*^ (0.02)	0.93^***^ (0.02)	0.75^***^ (0.03)	1.04 (0.03)	1.13^**^ (0.04)
South	1.81^***^ (0.07)	0.92^***^ (0.02)	0.92^***^ (0.02)	0.87^***^ (0.03)	1.13^***^ (0.03)	0.88^***^ (0.03)
Midwest	1.06 (0.04)	0.88^***^ (0.02)	0.87^***^ (0.02)	0.81^***^ (0.03)	1.11^***^ (0.03)	0.92^**^ (0.03)
West	1.00	1.00	1.00	1.00	1.00	1.00
Month						
April	1.00	1.00	1.00	N/A	N/A	N/A
May	0.99 (0.06)	1.09^**^ (0.03)	1.07^*^ (0.03)	N/A	N/A	N/A
June	1.06 (0.06)	1.11^***^ (0.03)	1.03 (0.03)	N/A	N/A	N/A
July	1.14^*^ (0.06)	1.06^*^ (0.03)	1.03 (0.03)	N/A	N/A	N/A
August	1.07 (0.05)	0.86^***^ (0.02)	0.75^***^ (0.02)	1.00	1.00	1.00
September	1.03 (0.05)	0.77^***^ (0.02)	0.68^***^ (0.02)	1.16^***^ (0.04)	1.03 (0.02)	1.04 (0.03)
October	1.09 (0.06)	0.72^***^ (0.02)	0.67^***^ (0.02)	1.18^***^ (0.04)	1.06^*^ (0.03)	1.17^***^ (0.04)
November	1.05 (0.06)	0.79^***^ (0.02)	0.69^***^ (0.02)	1.26^***^ (0.05)	1.15^***^ (0.03)	1.15^***^ (0.04)
December	1.09 (0.06)	0.86^***^ (0.02)	0.79^***^ (0.02)	1.43^***^ (0.05)	1.15^***^ (0.03)	1.19^***^ (0.04)

Logistic regression was used to estimate the association between access to care and income level, controlling for age, sex, race/ethnicity, education, marital status, housing tenure, region of residence, and survey month. ^***^p<0.001, ^**^p<0.01, ^*^p<0.05.

OR, odds ratio.

Table 3 provides the average predicted probabilities adjusting for full covariates. The average predicted probability of being uninsured was 12.2% for adults with household income less than $25,000 and 2.7% for those with income $200,000 and above. The difference in uninsured rates between these two income groups was 9.5 percentage points (12.2 percentage points—2.7 percentage points; *p*<0.001). The average predicted probabilities of delayed medical care due to the pandemic, delayed care of something other than COVID-19, and delayed mental health care were higher for adults with household income less than $25,000, compared to those with income $200,000 and above, by 9.1, 12.0, and 6.7 percentage points, respectively. The average predicted probability of taking prescription medication for mental health was higher for adults with household income less than $25,000, compared to those with income $200,000 and above by 10.2 percentage points. Although the average predicted probability of receiving mental health services was the highest for the lowest income group (12.52%), income groups between $25,000 and $199,999 presented a lower probability of receiving mental health services than those with income $200,000 and above (8.92–9.12% vs. 10.04%).

**Table 3.** Average Predicted Probabilities of Access to Care by Household Income Level Among U.S. Adults Aged 18 Years or Older, April–December 2020 Household Pulse Survey

	Uninsured rate	Delayed medical care due to pandemic	Delayed care of something other than COVID-19	Delayed mental health care	Taking prescription medication for mental health	Receiving mental health service
Sample size	674,381	673,352	673,803	366,399	366,228	366,183
Household income						
Less than $25,000	12.19 (0.29)	40.73 (0.49)	33.87 (0.47)	13.62 (0.38)	26.23 (0.50)	12.52 (0.38)
$25,000–$49,999	11.55 (0.22)	37.75 (0.33)	29.70 (0.30)	11.84 (0.25)	21.59 (0.32)	8.92 (0.22)
$50,000–$99,999	8.07 (0.18)	36.99 (0.26)	28.77 (0.25)	11.10 (0.21)	20.46 (0.23)	9.11 (0.17)
$100,000–$199,999	4.22 (0.18)	34.44 (0.30)	24.83 (0.27)	8.99 (0.23)	18.53 (0.27)	9.12 (0.19)
$200,000 and above	2.71 (0.30)	31.65 (0.43)	21.89 (0.39)	6.96 (0.33)	16.07 (0.41)	10.04 (0.38)
Missing	8.92 (0.40)	34.77 (0.60)	26.90 (0.55)	8.64 (0.43)	18.07 (0.55)	8.61 (0.44)
Age (years)						
18–24	10.17 (0.43)	32.22 (0.72)	23.71 (0.66)	17.19 (0.71)	19.16 (0.69)	11.84 (0.55)
25–34	13.76 (0.29)	36.32 (0.37)	27.35 (0.35)	15.25 (0.33)	19.70 (0.37)	12.76 (0.31)
35–44	12.12 (0.25)	38.98 (0.31)	30.42 (0.29)	13.68 (0.25)	22.05 (0.29)	12.65 (0.24)
45–54	10.39 (0.24)	39.77 (0.33)	32.38 (0.32)	11.19 (0.28)	23.32 (0.34)	10.16 (0.22)
55–64	7.35 (0.20)	38.51 (0.33)	30.30 (0.31)	7.88 (0.23)	21.94 (0.31)	7.52 (0.21)
65–74	1.05 (0.10)	33.84 (0.38)	24.93 (0.34)	4.49 (0.17)	18.65 (0.33)	5.01 (0.20)
75+	1.79 (0.32)	29.10 (0.59)	22.19 (0.56)	3.41 (0.46)	14.98 (0.60)	4.23 (0.51)
Sex						
Male	10.66 (0.16)	33.43 (0.22)	26.07 (0.20)	8.30 (0.17)	15.45 (0.20)	7.77 (0.15)
Female	7.62 (0.11)	39.58 (0.19)	30.31 (0.18)	12.87 (0.15)	25.08 (0.19)	11.15 (0.14)
Race/ethnicity						
Non-Hispanic White	8.09 (0.11)	37.16 (0.17)	28.27 (0.16)	11.54 (0.14)	23.78 (0.17)	10.49 (0.13)
Non-Hispanic Black	7.96 (0.24)	33.96 (0.48)	28.29 (0.45)	9.31 (0.34)	13.07 (0.41)	7.86 (0.29)
Hispanic	12.60 (0.29)	36.77 (0.46)	28.24 (0.43)	9.85 (0.32)	16.26 (0.42)	8.66 (0.30)
Non-Hispanic Asian	8.21 (0.47)	31.83 (0.60)	23.69 (0.56)	5.76 (0.32)	9.18 (0.44)	5.16 (0.31)
Non-Hispanic other race	7.65 (0.36)	41.31 (0.72)	34.68 (0.71)	13.57 (0.70)	21.35 (0.83)	10.67 (0.69)
Education						
Less than high school	14.37 (0.51)	31.55 (0.85)	25.79 (0.76)	8.94 (0.65)	18.25 (0.83)	7.83 (0.60)
High school	10.70 (0.20)	31.56 (0.32)	25.27 (0.30)	8.52 (0.24)	18.50 (0.31)	6.58 (0.21)
Some college	8.60 (0.14)	38.60 (0.22)	31.06 (0.20)	11.95 (0.17)	22.14 (0.20)	9.80 (0.15)
Bachelor's degree	5.62 (0.14)	39.36 (0.24)	28.29 (0.22)	11.87 (0.19)	20.97 (0.22)	11.62 (0.18)
Master's degree or higher	4.29 (0.16)	42.40 (0.27)	30.20 (0.26)	12.38 (0.24)	22.16 (0.25)	13.70 (0.23)
Marital status						
Currently married	7.50 (0.14)	36.77 (0.20)	28.51 (0.19)	9.16 (0.15)	19.37 (0.19)	8.10 (0.13)
Widowed	9.64 (0.84)	36.11 (0.77)	28.60 (0.73)	12.44 (0.87)	22.37 (0.69)	10.73 (0.67)
Divorced/separated	10.36 (0.25)	37.80 (0.38)	30.10 (0.36)	13.00 (0.32)	23.76 (0.37)	12.57 (0.32)
Never married	10.46 (0.19)	35.61 (0.34)	26.54 (0.31)	12.04 (0.26)	20.89 (0.37)	10.74 (0.27)
Missing	8.07 (1.41)	42.34 (2.35)	34.60 (2.29)	10.99 (1.41)	20.14 (2.01)	9.87 (1.36)
Housing tenure (home ownership)						
Renter	10.43 (0.16)	38.01 (0.29)	29.82 (0.27)	12.36 (0.22)	22.15 (0.29)	8.73 (0.12)
Owner	7.90 (0.14)	36.05 (0.18)	27.61 (0.17)	9.81 (0.14)	19.88 (0.17)	11.14 (0.21)
Missing	9.42 (0.66)	33.72 (1.35)	25.28 (1.27)	9.18 (1.34)	19.78 (1.24)	10.13 (1.03)
Region of residence						
Northeast	6.33 (0.21)	37.06 (0.36)	28.17 (0.34)	9.47 (0.25)	19.99 (0.32)	11.02 (0.25)
South	11.90 (0.17)	36.19 (0.23)	28.09 (0.22)	10.75 (0.18)	21.30 (0.23)	8.85 (0.17)
Midwest	7.77 (0.18)	35.33 (0.27)	27.08 (0.25)	10.14 (0.20)	21.03 (0.26)	9.22 (0.19)
West	7.41 (0.19)	38.13 (0.30)	29.72 (0.28)	12.06 (0.26)	19.35 (0.27)	9.94 (0.21)
Month						
April	8.68 (0.27)	38.57 (0.40)	31.39 (0.38)	N/A	N/A	N/A
May	8.57 (0.32)	40.57 (0.50)	32.80 (0.47)	N/A	N/A	N/A
June	9.09 (0.34)	40.93 (0.52)	31.97 (0.49)	N/A	N/A	N/A
July	9.63 (0.29)	40.01 (0.44)	32.05 (0.42)	N/A	N/A	N/A
August	9.20 (0.22)	35.23 (0.31)	25.72 (0.28)	9.16 (0.18)	19.42 (0.24)	8.75 (0.17)
September	8.89 (0.24)	32.66 (0.34)	23.97 (0.30)	10.41 (0.23)	19.88 (0.26)	9.04 (0.18)
October	9.31 (0.28)	31.31 (0.37)	23.61 (0.34)	10.56 (0.26)	20.27 (0.31)	10.01 (0.24)
November	9.02 (0.31)	33.37 (0.44)	24.10 (0.40)	11.17 (0.27)	21.57 (0.34)	9.91 (0.25)
December	9.31 (0.28)	35.06 (0.41)	26.69 (0.39)	12.40 (0.30)	21.64 (0.34)	10.15 (0.26)

Average adjusted predictions were computed after logistic regression estimation controlling for full list of covariates. All predictive margins were statistically significant at *p*<0.001.

## Discussion

Our study contributes to the existing literature by estimating the monthly trend of various access measures across income groups and by adding the evidence on the association between access to care and household income level among adults aged ≥18 years, using a nationally representative dataset from April to December during the COVID-19 pandemic in the United States. We found significantly higher adjusted odds of being uninsured, delayed care due to pandemic, delayed care of something other than COVID-19, and delayed mental health care among individuals with lower household incomes than those with higher household incomes. These results differ from findings of two recent reports that the delayed care during the pandemic was less likely among low-income adults^2^ or had no association with income level.^3^ Given that both reports used surveys from June 2020, and we found that the delayed medical care varied by month, differences in the findings might stem from the analysis of different time periods of the survey.

The odds of receiving mental health services were also higher for lower income groups than those for higher income groups, except the lowest income group (less than $25,000). We found that the odds of taking prescription medication for mental health were higher for lower income groups than those for higher income groups. The higher odds of receiving mental health services among adults with household income less than $25,000 and taking prescription medication for mental health for lower income groups might be explained by their increased vulnerability to mental distress due to job insecurity and financial concerns during the pandemic.^24,25^

Further studies are needed to focus on delayed access to specialty care such as cancer,^13,26^ chronic pain,^11^ and acute cares^7^ or self-reported reasons of delayed care^2^ by income level or race/ethnicity. Recent studies found that cancer screenings, visits, therapy, and surgeries have significantly decreased and surgery for chronic pain were deferred during the pandamic.^11,13,26^ Given our findings, access to specialty care might also differ by household income level. Moreover, considering that utilization of health care services and health status were associated with neighborhood-level socioeconomic characteristics or racial/ethnic composition,^27^ supply of physician or specialty care medical professionals,^28^ or area deprivation,^29^ it would be worthwhile to examine the association between access to care during the pandemic with neighborhood-level characteristics.

To improve access to care during the COVID-19 pandemic, telehealth might be an alternative solution for individuals to obtain necessary medical care.^30–33^ Policymakers should make an effort on removing barriers to the use of telehealth such as reimbursement or technology infrastructure.^30^ For example, the Centers for Medicare and Medicaid Services (CMS) extended the reimbursement for remote patient monitoring services for acute and chronic conditions during the public health emergency.^31^ However, the equitable access to telehealth should be established given socioeconomic and racial/ethnic inequalities in access to care.^32^ The CMS report found that beneficiaries with income more than $25,000 were more likely to have access to telemedicine appointments compared with those with income below $25,000.^5,6^ Clinical training and guidance for health care professionals are needed to increase skilled workforce who can switch delivery modes to telehealth.^30,33^

### Limitations

This study has limitations. First, since we used cross-sectional survey data, the estimated associations between income and access to care do not infer causality. However, since household income was measured as of 2019 but access to care in HPS was measured during the pandemic since March 2020, the estimates were less likely to be affected by reverse causality. Second, we did not control for state-level or county-level covariates, such as state's decision on Medicaid expansion, or county-level household income, unemployment rate, or physician supply.^15^ Low-income individuals who reside in states, which opted out of the expansion of Medicaid eligibility, could have less access to medical care, compared with expansion states.^34,35^ Third, the timing of delayed care could be lagged since respondents were asked their experience in last 4 weeks preceding the survey. For example, week 1 data collected from April 23 to May 5 could include delayed care experienced in late March or early May. For consistency and comparability, we considered data collected during a month as the timing of delayed care in the trend graphs. Fourth, the respondents in HSP are more likely to be women and non-Hispanic whites and have a higher education, compared with the American Community Survey.^24^ Overrepresentation of non-Hispanic whites and those with higher education might result in an underestimate of the magnitude of income disparities in uninsured rates, delayed care, or health care utilization. We addressed disproportionate sampling of demographic characteristics by using survey weights, which rakes the demographics of the interviewed persons to education attainment/sex/age distributions and ethnicity/race/sex/age population distributions.^19^

## Conclusions

Using the nationally representative survey data from April to December 2020, we found a strong association between access to care and household income level among adults aged ≥18 years during the COVID-19 pandemic. Specifically, the likelihood of being uninsured rate and having delayed any medical care, care of something other than COVID-19, and mental health care was significantly higher for adults with lower household incomes than those with higher incomes. While the odds of taking prescription medication for mental health were higher for adults with low household incomes than those with high income, the odds of receiving mental health services were lower for lower income groups, except for the lowest income group, controlling for all covariates. During the pandemic, U.S. adults with lower household incomes had statistically significantly higher rates of uninsurance, delayed care, and the use of mental health services than those with higher income. These findings emphasize the need for increased policy efforts to improve access to care among low-income adults and households to reduce health care inequities during the ongoing pandemic and beyond.

## Abbreviations Used

CMS: Centers for Medicare and Medicaid Services
COVID-19: coronavirus disease 2019
HPS: Household Pulse Survey
OR: odds ratio
SE: standard error
